# Editorial: Genetic and immunological insights into angioedema without wheals

**DOI:** 10.3389/fimmu.2026.1787741

**Published:** 2026-01-29

**Authors:** Matija Rijavec, Maurizio Margaglione, Anastasios E. Germenis

**Affiliations:** 1 University Clinic of Respiratory and Allergic Diseases Golnik, Golnik, Slovenia; 2 Biotechnical Faculty, University of Ljubljana, Ljubljana, Slovenia; 3 Faculty of Medicine, University of Maribor, Maribor, Slovenia; 4 Medical Genetics, Department of Clinical and Experimental Medicine, University of Foggia, Foggia, Italy; 5 Department of Immunology & Histocompatibility, School of Health Sciences, Faculty of Medicine, University of Thessaly, Larissa, Greece

**Keywords:** angioedema, biomarker, bradykinin, genetic, inflammatory mediators, outcome, real-world data, treatment

Angioedema without wheals (AE) is a distinct clinical entity characterized by recurrent, self-limiting episodes of localized swelling occurring in the absence of urticaria. Attacks may affect the face, lips, tongue, larynx, gastrointestinal tract, genitalia, or extremities, and typically do not respond to antihistamines or corticosteroids, necessitating interventions that primarily target the bradykinin pathways. Despite a shared clinical phenotype, AE reflects diverse underlying molecular mechanisms, creating persistent diagnostic and therapeutic challenges across emergency medicine, allergy/immunology, internal medicine, and genetics settings. Misdiagnosis and delayed recognition remain common, prolonging avoidable morbidity and, in the case of laryngeal involvement, exposing patients to potentially fatal risk.

This Research Topic brings together 12 contributions spanning mechanistic hypotheses, biomarker discovery, genetic insights, real-world treatment evidence, health-service delivery, and patient-centred outcomes. Taken together, these studies convey a clear unifying message: sustained progress in AE arises from aligning biology (genetics, inflammatory mediators, and pathway crosstalk) with measurement (robust, validated outcomes and emerging digital monitoring approaches) and implementation (pragmatic prophylaxis strategies and models of care).

## Bridging pathways: from bradykinin to inflammation

A central challenge in AE is understanding why clinically similar swelling phenotypes can arise from different upstream drivers (e.g., contact pathway dysregulation in hereditary angioedema (HAE) vs. mast-cell–mediated processes), and why triggers such as stress or intercurrent inflammation can precipitate attacks. Porebski et al. revisit one of the field’s most compelling unresolved questions: whether, and by what mechanisms, mast cell degranulation might intersect with bradykinin-driven AE, proposing candidate mechanistic links and highlighting knowledge gaps that must be addressed experimentally. Two original research contributions add depth to the inflammation story. Gil-Serrano et al. examine systemic inflammation biomarkers during acute HAE attacks, leveraging a multi-marker approach that aims to distinguish baseline from attack-associated inflammatory signatures and to refine biomarker candidates for future validation, suggesting that inflammation extends beyond the localized edematous area. In a complementary direction, use a controlled stress paradigm, the socially evaluated cold pressor test, to quantify stress reactivity in HAE-C1INH. They report higher perceived stress and elevated cytokine levels (notably IL-6 and TNF-α patterns) in patients compared with controls, supporting a biologically plausible link between stress, inflammatory mediators, and disease vulnerability.

## Genetics, interpretation, and comorbidity: improving diagnostic precision

As genetic testing becomes increasingly integrated into routine practice, the field faces a new dilemma: how to interpret and responsibly communicate results that are incidentally discovered, especially in HAE with normal C1 inhibitor (HAE-nC1INH), where genetic confirmation is often required for accurate diagnosis. Germenis and Sanoudou provide a timely practice-oriented framework for handling incidental findings and variants of uncertain significance in genes associated with HAE-nC1INH, arguing for restraint when evidence on penetrance and prevalence is insufficient, and for structured collaboration among clinicians, laboratories, and genetic counsellors when results are clinically ambiguous. This guidance is particularly important in AE, where misclassification can lead to inappropriate treatment selection, unnecessary investigations, and avoidable patient anxiety. Beyond genetics, comorbid autoimmune disease can further complicate clinical presentation, confound biomarker interpretation, and influence longitudinal management. Triggianese et al. provide the first large-cohort evidence on the prevalence and spectrum of rare connective tissue diseases in HAE-C1INH, identifying systemic lupus erythematosus as the most frequent diagnosis. These observations highlight the importance of clinical awareness and, when appropriate, focused assessment supported by coordinated multidisciplinary care.

## Measuring what matters: disease activity, disease control, sleep, and patients’ experience

An enduring limitation in AE research has been the mismatch between clinician-centred endpoints and patient experience. Heibati et al. directly address this issue by evaluating the relationship between the Angioedema Control Test (AECT) and the Hereditary Angioedema Activity Score (HAE-AS) in routine care. Their finding of only a weak correlation supports a pragmatic conclusion: disease activity and disease control are not interchangeable, and using both tools can provide a more comprehensive understanding of the clinical picture and guide more informed treatment decisions. Quality-of-life impairments extend well beyond the number of attacks. Karabiber et al. demonstrate that sleep disruption is prevalent in HAE and worsens during attacks across anatomical locations, with clinically meaningful increases in subjective sleep disturbance measures, reinforcing the need to screen for sleep problems and treat them as part of comprehensive disease management rather than as secondary concerns. Parati et al. evaluate a 16-week ecological momentary assessment (EMA) protocol for affect monitoring in HAE, reporting strong feasibility and acceptability (high recruitment and completion rates, brief completion time, and favorable participant ratings). Together with the stress-reactivity study, this work suggests a realistic pathway to integrating psychological context and biological measures, an essential step if we aim to understand triggers, anticipate attacks, and personalize supportive interventions.

## From mechanism to management: prophylaxis strategies and care delivery in the real world

Effective therapy for AE without wheals and bradykinin-mediated AE has expanded rapidly, but implementation remains uneven across health systems. Srinivasan et al. present real-world Canadian data on berotralstat for long-term prophylaxis, describing reductions in attack frequency alongside generally manageable gastrointestinal adverse effects and favorable treatment satisfaction. Adatia and Magerl complement this with a clinician-facing practical guide, summarizing pharmacology, tolerability strategies (including graded introduction protocols), drug–drug interactions, and considerations for switching and overlap between long-term prophylaxis agents. Access and cost constraints remain a reality in many settings, underscoring the importance of evidence on older therapies and system-level solutions. Ritter et al. provide real-world insights into the use of danazol for HAE-C1INH in the Brazilian public health system, informing risk–benefit discussions where modern biologics or newer small molecules may be less available. Finally, Holmes et al. describe how a centralised care model can overcome geographic barriers, which is highly relevant for rare diseases where expertise is concentrated and delayed diagnosis is common.

## Looking ahead

The evidence gathered in this Research Topic points to three immediate priorities. First, we need validated biomarkers that improve diagnosis and enable biologically meaningful stratification across AE phenotypes in both attack and remission states. Second, outcomes should be captured in a multi-dimensional way, combining disease activity, control, sleep and patient-reported burden, so that clinical trials and routine care reflect what patients experience. Third, advances must be translated through implementation-focused efforts, including scalable care pathways, equitable access to prophylaxis, and practical frameworks for switching, monitoring, and long-term follow-up in real-world settings. AE without wheals remains a high-impact diagnostic and therapeutic challenge, but the field is moving toward a more integrated, patient-centred model in which genetics, immunology, and patient-centred care delivery evolve together.

